# Comparison between the open and the laparoscopic approach in the primary ventral hernia repair: a systematic review and meta-analysis

**DOI:** 10.1007/s00423-024-03241-y

**Published:** 2024-02-03

**Authors:** Márcia Regina Martins, Hugo Santos-Sousa, Miguel Alves do Vale, Raquel Bouça-Machado, Elisabete Barbosa, Bernardo Sousa-Pinto

**Affiliations:** 1 Faculty of Medicine, University of Porto, Porto, Portugal; 2 Integrated Responsibility Center for Obesity (CRIO), São João University Medical Centre, Alameda Professor Hernâni Monteiro, 4200-319 Porto, Portugal; 3 Institute of Molecular Medicine João Lobo Antunes, Lisbon, Portugal; 4 Department of Surgery, São João University Medical Centre, Porto, Portugal; 5 MEDCIDS – Department of Community Medicine, Information and Health Decision Sciences, Faculty of Medicine, University of Porto, Porto, Portugal; 6 CINTESIS – Centre for Health Technologies and Services Research, University of Porto, Porto, Portugal

**Keywords:** Ventral hernia, Umbilical hernia, Paraumbilical hernia, Epigastric hernia, Laparoscopic repair, Open repair

## Abstract

**Background:**

Ventral hernia repair underwent various developments in the previous decade. Laparoscopic primary ventral hernia repair may be an alternative to open repair since it prevents large abdominal incisions. However, whether laparoscopy improves clinical outcomes has not been systematically assessed.

**Objectives:**

The aim is to compare the clinical outcomes of the laparoscopic versus open approach of primary ventral hernias.

**Methods:**

A systematic search of MEDLINE (PubMed), Scopus, Web of Science, and Cochrane Central Register of Controlled Trials was conducted in February 2023. All randomized controlled trials comparing laparoscopy with the open approach in patients with a primary ventral hernia were included. A fixed-effects meta-analysis of risk ratios was performed for hernia recurrence, local infection, wound dehiscence, and local seroma. Meta-analysis for weighted mean differences was performed for postoperative pain, duration of surgery, length of hospital stay, and time until return to work.

**Results:**

Nine studies were included in the systematic review and meta-analysis. The overall hernia recurrence was twice less likely to occur in laparoscopy (RR = 0.49; 95%CI = 0.32–0.74; *p* < 0.001; *I*^2^ = 29%). Local infection (RR = 0.30; 95%CI = 0.19–0.49; *p* < 0.001; *I*^2^ = 0%), wound dehiscence (RR = 0.08; 95%CI = 0.02–0.32; *p* < 0.001; *I*^2^ = 0%), and local seroma (RR = 0.34; 95%CI = 0.19–0.59; *p* < 0.001; *I*^2^ = 14%) were also significantly less likely in patients undergoing laparoscopy. Severe heterogeneity was obtained when pooling data on postoperative pain, duration of surgery, length of hospital stay, and time until return to work.

**Conclusion:**

The results of available studies are controversial and have a high risk of bias, small sample sizes, and no well-defined protocols. However, the laparoscopic approach seems associated with a lower frequency of hernia recurrence, local infection, wound dehiscence, and local seroma.

## Introduction

A hernia is a protrusion of tissue or an organ through an abnormal opening that can be primary or acquired (for example at the site of a previous surgical incision—incisional hernia) [1, 2]. Hernias can occur in various anatomic locations, the most common being inguinal hernias [1] followed by ventral hernias [3].

Abdominal wall hernias can be classified into primary ventral and incisional hernias [4]. Over 300,000 and 350,000 ventral hernia repairs are performed annually in Europe and the USA, respectively. Of these, approximately 75% are due to primary defects (mainly epigastric, umbilical, paraumbilical, and Spigelian hernias) and 25% are due to incisional hernias [5].

Nevertheless, while both incisional and primary ventral hernias are commonly grouped, it is necessary to consider that each has a distinct pathogenesis, different patient risk factors, and therefore often different therapeutic strategies [6].

Hernias can cause pain and discomfort that can significantly impact the quality of life of the patients. Moreover, they may lead patients to have a negative association with body image and to serious complications such as bowel incarceration [2, 6]. Therefore, ventral hernias are usual indications for surgery and should be corrected.

Treatment of abdominal wall hernias is a rapidly evolving field of surgery, given the dramatic rise in the number of laparotomies and major surgeries being performed, the progress in anesthesiology, the increase in the number of older patients with weak connective tissue, and the increased prevalence of risk factors for hernias [7].

Several studies showed that the costs of surgery in laparoscopic ventral hernia repair were higher when compared with the open approach because it normally requires more expensive mesh types. However, laparoscopic repair seems to be associated with fewer complications, shorter duration of hospital stay, fewer readmissions, fewer outpatient appointments, and fewer days off work than open repair. These findings can reduce post-treatment costs and might make this type of surgery more cost-efficient in comparison to open surgery [2, 8].

The laparoscopic approach involves minimally invasive access to the abdominal cavity, and a prosthesis can be placed deep into the abdominal fascia typically without the disturbance of the hernia sac. This technique reduces the surgical insult and provides an improved view of the defect, including smaller defects that may not be identified during the clinical examination. As a result, this facilitates accurate placement of the prosthesis with reliable fascial overlap. Furthermore, it can also help to minimize the risk of bleeding, seroma formation, bowel wall injury, and infectious complications [9].

Nevertheless, to the best of our knowledge, there are no systematic reviews only evaluating primary ventral hernia repairs, and the results of available studies and subgroup analysis remain somehow controversial, especially regarding the duration of surgery.

The primary objective of this systematic review is to compare the clinical results of the laparoscopic approach compared with the open approach of primary ventral hernias, specifically epigastric, umbilical, and paraumbilical hernias.

## Methods

This systematic review and meta-analysis was executed in conformity with the “Preferred Reporting Items for Systematic Reviews and Meta-Analyses” (PRISMA) guidelines [10].

### Eligibility criteria for considering studies for this review

In this systematic review, we included all randomized controlled trials that compared the laparoscopic with the open approach in patients aged ≥ 18 years with a primary ventral hernia (specifically epigastric, umbilical, and paraumbilical hernias) who were submitted to elective repair. Studies were comprised regardless of the type of surgery, mesh type, material, placement, or method of fixation. Studies were included regardless of the year of publication, language, publication status, or sample size.

Studies that included patients with a recurrent hernia, incisional hernia, Spigelian hernia, lumbar hernia, acute or subacute intestinal obstruction, abdominal malignancies, or ascites were excluded. Patients who were submitted to emergency surgery or had more than one simultaneous surgery (for example, bariatric surgery with concomitant hernia repair) were, also, excluded.

### Search method

A systematic search of MEDLINE (PubMed), Scopus, Web of Science, and Cochrane Central Register of Controlled Trials was conducted in February 2023 using the search strategies displayed in Table 1. No filters or limits were used. Furthermore, an assessment of reference bibliographies from included primary studies was performed.

**Table 1:** Literature search queries

Databases	Queries
MEDLINE (PubMed)	(“hernia, ventral”[MeSH] OR (hernia*[Title/Abstract] AND (ventral[Title/Abstract] OR incisional[Title/Abstract] OR epigastric[Title/Abstract] OR umbilical[Title/Abstract] OR parastomal[Title/Abstract] OR spiegel*[Title/Abstract] OR spigel*[Title/Abstract]))) AND ("laparoscopy"[ MeSH] OR laparosc*[Title/Abstract])
Scopus	( TITLE-ABS-KEY ( “hernia”) AND ( TITLE-ABS-KEY ( “ventral”) OR TITLE-ABS-KEY ( “incisional”) OR TITLE-ABS-KEY ( “epigastric”) OR TITLE-ABS-KEY ( “umbilical”) OR TITLE-ABS-KEY ( “parastomal”) OR TITLE-ABS-KEY ( “spiegel*”) OR TITLE-ABS-KEY ( “spigel*”))) AND ( TITLE-ABS-KEY ( “laparoscopy”) OR TITLE-ABS-KEY ( “laparosc*”))
Web of Science	(ALL = (“hernia”) AND ( ALL = (“ventral”) OR ALL = (“incisional”) OR ALL = (“epigastric”) OR ALL = (“umbilical”) OR ALL = (“parastomal”) OR ALL = (“spiegel*”) OR ALL = (“spigel*”))) AND (ALL = ("laparoscopy") OR ALL = (“laparosc*”))
Cochrane Central Register of Controlled Trials	(“hernia, ventral”[Mesh] OR (hernia*[ti,ab,kw] AND (ventral[ti,ab,kw] OR incisional[ti,ab,kw] OR epigastric[ti,ab,kw] OR umbilical[ti,ab,kw] OR parastomal[ti,ab,kw] OR spiegel*[ti,ab,kw] OR spigel*[ti,ab,kw]))) AND ("laparoscopy"[Mesh] OR laparosc*[ti,ab,kw])

### Selection of studies

The title and the abstract of all the studies identified by the search strategy were independently screened for potential eligibility by two reviewers (MM and MV). Disagreements were solved by meeting and debating with a third reviewer (HS) to reach a consensus. Subsequently, the full texts of articles not excluded in the previous stage were thoroughly independently reviewed by the same reviewers (MM and MV) and checked against the inclusion criteria. When different articles corresponding to the same study were found, only the latest was included.

### Data collection

Data collection was executed by one reviewer (MM) and checked by a second reviewer (MV). Data extracted from the studies consisted of the study design, sample size, description of the surgery approach, duration of surgery, follow-up period, and outcomes measured. The main assessed outcome was hernia recurrence. Additional outcomes included the duration of surgery, length of hospital stay, time until return to work, and surgery complications such as seroma, wound dehiscence, local infection, and postoperative pain. The collected data were entered and managed in RevMan 5 Software [11].

In the presence of incongruencies and missing data in primary studies, we contacted the authors to try to obtain the correct information. If that was not possible, the data was collected and analyzed according to the description of the methods and results given by the authors, not by the statistical analysis.

### Risk of bias assessment

The quality (risk of bias (RoB)) of included studies was independently evaluated by two reviewers (MM and MV) according to the Cochrane RoB2 tool regarding the randomization process, intended intervention, missing outcome data, measurement of the outcome, and selection of the reported result [12, 13]. Subsequently, the data from the evaluation of bias was summarized using the robvis tool [14].

### Data analysis

All the results that were measured on the same scale or could be converted to the same units were included in the meta-analysis. For studies that evaluated a determined outcome more than once only the latest evaluation was taken into account, for example, if a study evaluated pain at 2 h and 24 h post-surgery, only the 24-h measure was considered.

Furthermore, if studies compared more than one type of laparoscopic approach with the open approach, we independently analyzed both techniques with the open approach.

Risk ratios (RR) were calculated for all the dichotomous outcomes using 95% confidence intervals (CI). Continuous outcomes reported in the parametric form (mean with standard deviation) were evaluated and presented as weighted mean differences (MD). If standard deviations for continuous outcome data were not available, we estimated its value from the standard error of the mean, when feasible.

The *I*^2^ statistic was calculated to quantify the heterogeneity. An *I*^2^ inferior to 25% corresponded to minimal or no heterogeneity, an *I*^2^ between 25 and 50% was related to mild to moderate, an *I*^2^ within 50 to 75% correlated to moderate to substantial, and an *I*^2^ superior to 75% was associated with substantial to maximum heterogeneity [15]. In the presence of substantial heterogeneity (*I*^2^ > 50% and *p*-value < 0.10) we performed a random-effects meta-analysis. Otherwise, the fixed-effects model was used.

A leave-one-out sensitivity analysis was performed when the heterogeneity was substantial. Graphical display by funnel plots was used to evaluate the presence of publication bias. Meta-regression and subgroup analysis were not performed on account of the small number of primary studies included.

## Results

### Study selection

**Fig. 1:**
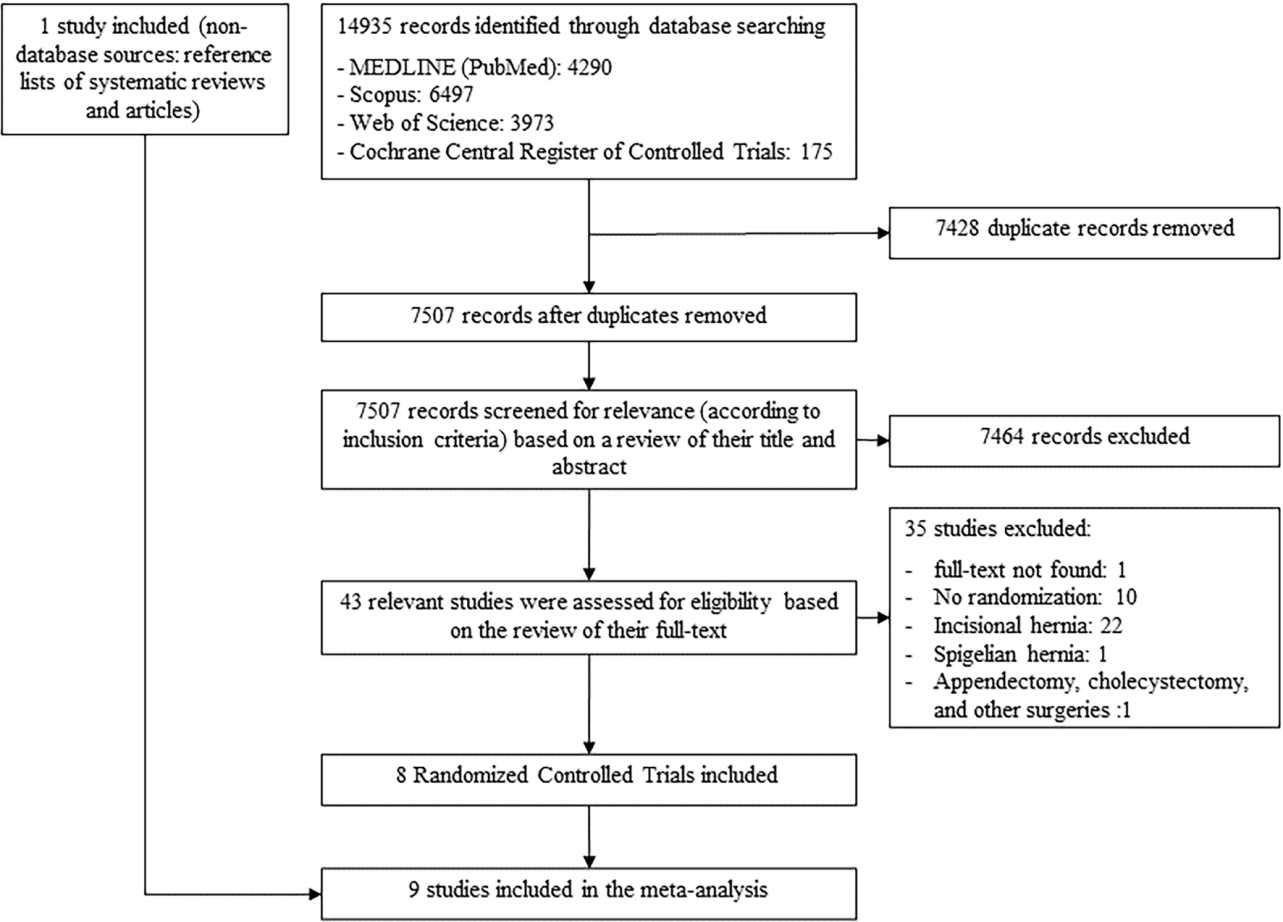
Flow diagram for the researched studies (February 2023). A total of 14,936 studies were identified although only 9 studies were included in the final analysis. The other studies were excluded due to ineligibility

The initial search identified 14935 possible records from which 7428 were duplicates. The remaining 7507 unique citations were reviewed by their title and abstract and 7464 were excluded for not meeting the inclusion criteria. In this manner, a total of 43 studies were identified as potentially eligible, and a full analysis of their text was made. Thirty-five studies were excluded: one, because we could not have access to their full text in the absence of authors’ response to our contact [16], ten for having a non-randomized design [17–26], one for comparing the open and laparoscopic approach grouping multiple surgeries (ventral hernia repair, appendectomy, cholecystectomy, and bariatric surgery) [27], and twenty-three for including all kinds of ventral hernias: one Spigelian hernia [28] and twenty-two incisional hernias from which thirteen only included incisional hernias [29–41] and nine included incisional and primary ventral hernias combined in the analysis [42–50]. One study was included after the assessment of the references of included primary studies [51]. Therefore, nine randomized controlled trials met the inclusion criteria and were included in this systematic review and meta-analysis [51–59]. The details of the selection process are displayed in Fig. 1.

### Study characteristics

Table 2 provides a descriptive summary of the characteristics of the included studies. The included studies were published between 2012 and 2022 and were conducted in Egypt [52], Pakistan [53–56, 58, 59], and India [51, 57]. All of the studies were randomized controlled trials, four studies were monocentric [51–53, 55] while the rest were multicentric studies [54, 56–59].

**Table 2:** Characteristics of included studies

Author, year	Study methodology	Participants (N)	Interventions		Outcomes	Follow-up duration
			Laparoscopic repair (*N*)	Open repair (*N*)		
Elashry et al. (2022) [52]	Monocentric randomized controlled trial	Paraumbilical hernia and defect diameter < 8 cm (60)	IPOM plus (28)	Onlay mesh repair with closure of defect by prolene sutures (18)	Pain, wound infection, seroma, recurrence rate, hospital stay, mesh size, postoperative drains, operative time, estimated blood loss, time off work, and cost of surgery	Not reported
			IPOM (14)			
Kashif et al. (2020) [53]	Monocentric randomized controlled trial	Paraumbilical hernia (180)	Not specified (90)	Not specified (90)	Operative time, hospital stay, wound infection, wound dehiscence, and recurrence	6 months
Khan et al. (2012) [54]	Multicentric randomized controlled trial	Ventral abdominal hernia (umbilical hernia, paraumbilical hernia, epigastric hernia) and defect diameter < 3 cm (100)	IPOM (50)	Onlay mesh repair with closure of defect by prolyglactin sutures (50)	Duration of surgery, postoperative pain score at 2 h, postoperative pain score at 24 h, and postoperative hospital stay	Not reported
Maaz-Ul-Hassan & SHAHAB (2019) [55]	Monocentric randomized controlled trial	Paraumbilical hernia (200)	Not specified (100)	Not specified (100)	Operative time, hospital stay, wound infection, and recurrence	6 months
Malik (2015) [56]	Multicentric randomized controlled trial	Paraumbilical hernia (337)	IPOM (166–11)	Preperitoneal mesh repair with the closure of defect by non-absorbable suture (171 + 11)	Duration of the surgery, prolonged ileus, hematoma, intestinal injury, seroma, bleeding during the adhesiolysis, cellulitis of trocar site, wound/mesh infection, prolonged pain (> 4 months), wound dehiscence, port herniation, recurrent hernia and length of hospital stay	2 years
Purushotham & Madhu (2015) [51]	Monocentric randomized controlled trial	Umbilical and paraumbilical hernia (42)	IPOM (21)	Infra-umbilical smile incision and mesh of appropriate size (21)	Operative time, postoperative pain at 6 h, postoperative pain at 24 h, hospital stay, return to work, return to daily activities, wound infection, seroma, recurrence, and cost factor analysis	Not reported. In some outcomes, a follow-up of 3 months is reported
Saniya et al. (2022) [57]	Multicentric randomized controlled trial	Umbilical hernia < 3 cm (80)	Not specified (40)	Not specified (40)	Swelling, swelling and pain, duration of the procedure, postoperative pain, seroma, wound infection, and duration of hospital stay	12 weeks
Shah et al. (2021) [58]	Multicentric randomized controlled trial	Ventral abdominal hernia (umbilical hernia, paraumbilical hernia, epigastric hernia) (190)	IPOM (95)	Onlay mesh repair (95)	Hospital stay, operative time, wound infection, wound dehiscence, and recurrence	12 months
Ul Hassan et al. (2019) [59]	Multicentric randomized controlled trial	Paraumbilical hernia (350)	IPOM (171–12)	Preperitoneal mesh repair with the closure of defect by non-absorbable suture (179 + 12)	Duration of the surgery, prolonged ileus, hematoma, intestinal injury, seroma, bleeding during the adhesiolysis, cellulitis of trocar site, wound/mesh infection, prolonged pain (> 4 months), wound dehiscence, port herniation, recurrent hernia, and length of hospital stay	2 years

All studies had similar inclusion and exclusion criteria and assessed similar outcomes. However, the methods used to assess the outcomes were, in some cases, different. Two studies included patients with epigastric, umbilical, and paraumbilical hernias [54, 58], one included patients with umbilical or paraumbilical hernias [51], one only included umbilical hernias [57], and the remaining five studies only included paraumbilical hernias [52, 53, 55, 56, 59].

A total of 1539 patients were included in the systematic review and meta-analysis, 775 were randomized to the laparoscopic repair group, and 764 were randomized to the open repair group with a follow-up period between 3 months and 2 years. In two studies, some patients had their laparoscopic repair converted into an open repair and were analyzed as treated [56, 59]. Regarding the surgical procedure, two studies did not specify or describe the techniques used for the laparoscopic or open approach [53, 57].

### Risk of bias in included studies

**Fig. 2:**
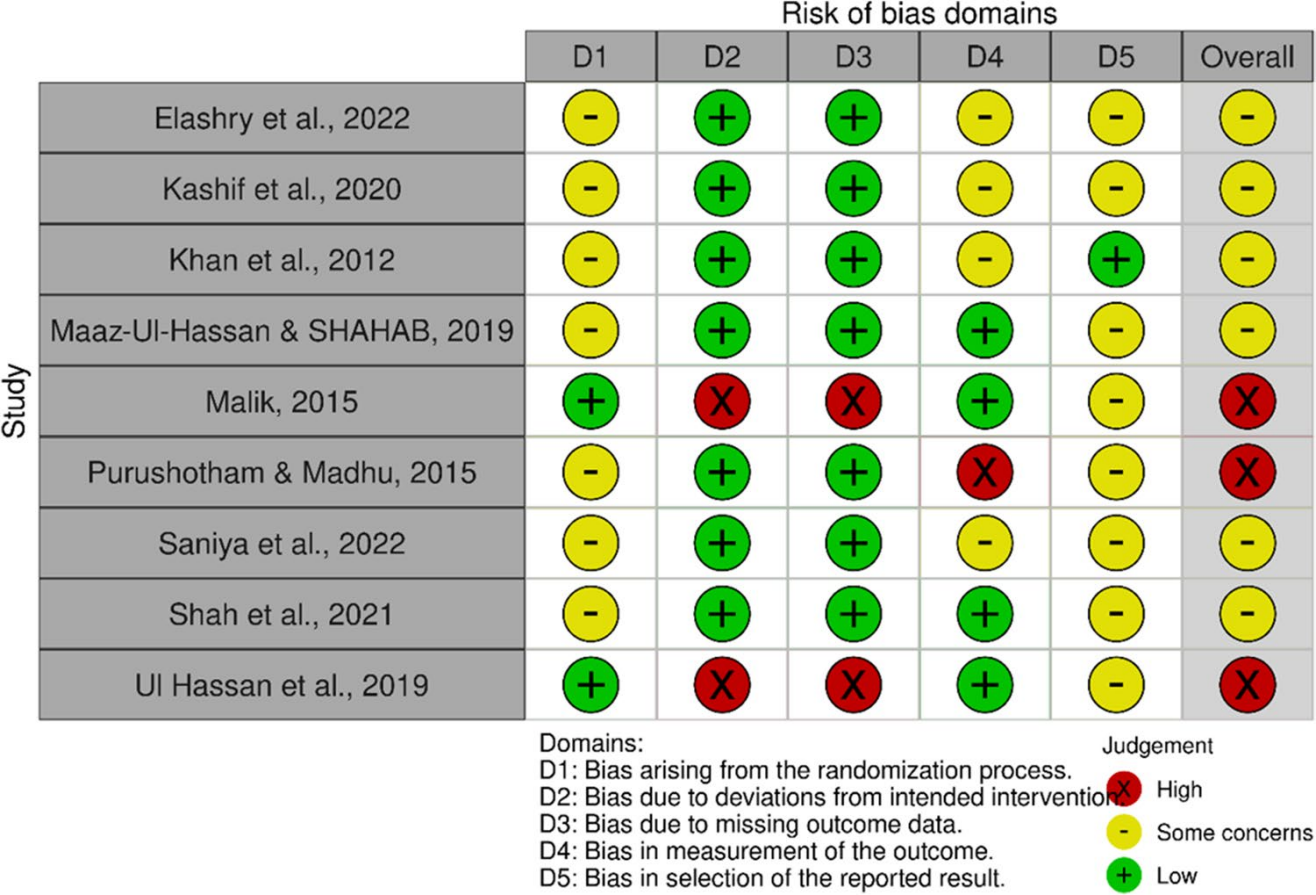
Methodological quality summary using the RoB2 and robvis tools. Assessment of the risk of bias in the included studies

**Fig. 3:**
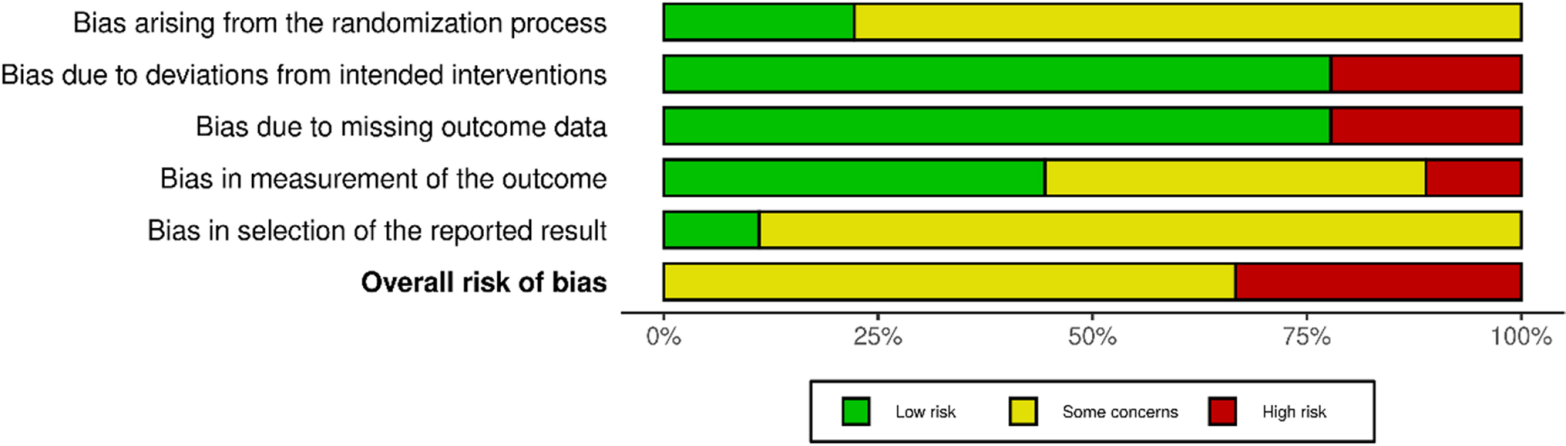
Methodological quality graph using the RoB2 and robvis tools. Assessment of each methodological quality item across all studies

The quality of the included studies was medium to high (Figs. 2 and 3). A total of three studies were evaluated as high risk of bias [51, 56, 59]. The exact method of allocation was only mentioned in three studies [54, 56, 59], and none of the studies had information about the blinding application. Two studies had incongruences in the data, missing data, and the analysis was not made following the intention-to-treat principle [56, 59].

Additionally, none of the studies defined a primary outcome measure that allowed the calculation of the sample size or mentioned having done a formal calculation of the sample size. The methods for evaluating a particular outcome were, in the majority of the cases, not determined.

### Effects of interventions

In the interpretation of the results, it is important to take into consideration the high risk of bias in some studies [56, 59] and that the results may be associated with some information or indication bias.

#### Hernia recurrence

**Fig. 4:**
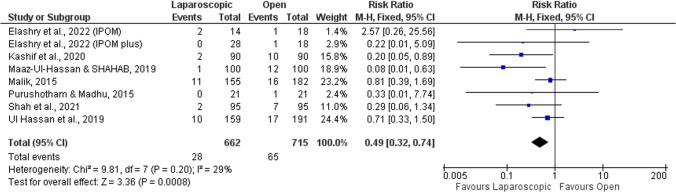
Forest plot of hernia recurrence

Seven studies evaluated the recurrence of hernia [51–53, 55, 56, 58, 59]. Recurrence rates were significantly different and found to be twice less likely to occur after laparoscopic than open repair (RR = 0.49; 95%CI = 0.32–0.74; *p* < 0.001; Fig. 4). However, the follow-up time was not ideal, less than 2 years in most of the studies, which could justify the small number of hernia recurrences in some studies. Furthermore, there was mild heterogeneity (*I*^2^ = 29%) which might affect the interpretation of the summary estimate.

#### Local infection

**Fig. 5:**
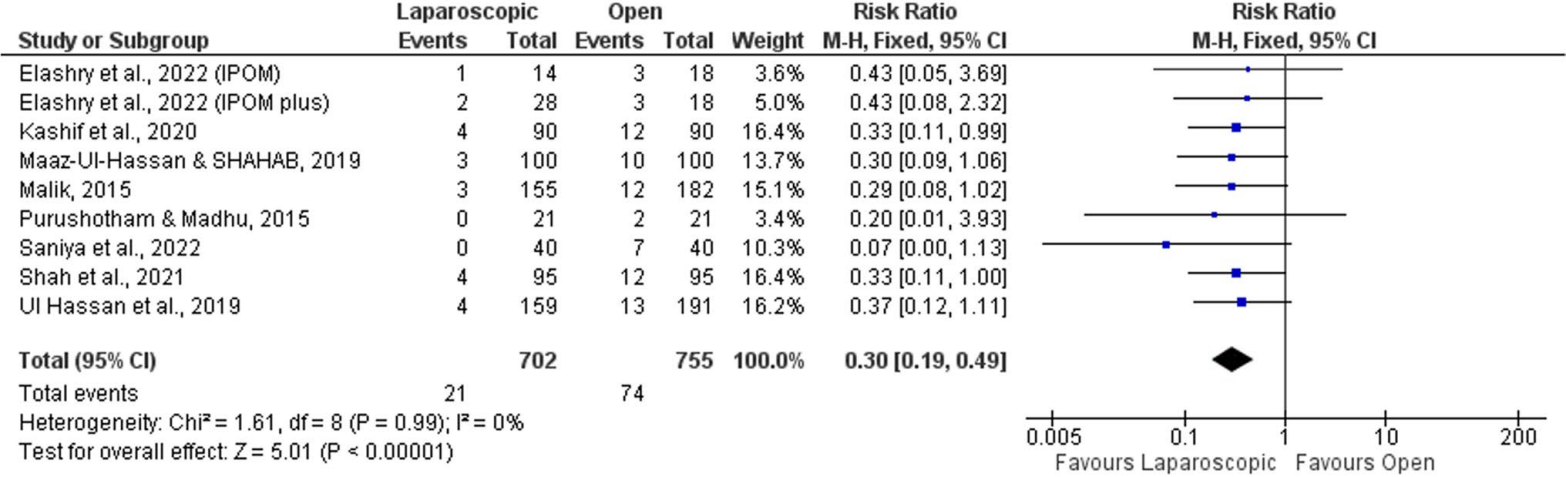
Forest plot of local infection

A total of eight studies evaluated the occurrence of local infection [51–53, 55–59]. Regarding this outcome, the laparoscopic approach showed significant benefits when compared with the open approach (RR = 0.30; 95%CI = 0.19–0.49; *p* < 0.001; Fig. 5), with no heterogeneity detected across the studies (*I*^2^ = 0%).

#### Wound dehiscence

**Fig. 6:**
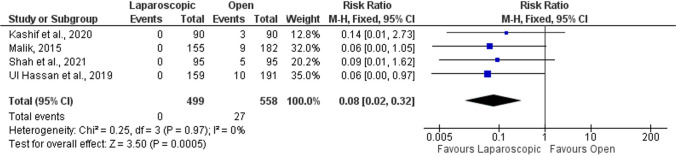
Forrest plot of wound dehiscence

Wound dehiscence was reported in four studies [53, 56, 58, 59]. The analysis indicated that wound dehiscence was 92% less likely to happen after laparoscopic than open repair (RR = 0.08; 95%CI = 0.02–0.32; *p* < 0.001; Fig. 6), with no heterogeneity detected (*I*^2^ = 0%).

#### Local seroma

**Fig. 7:**
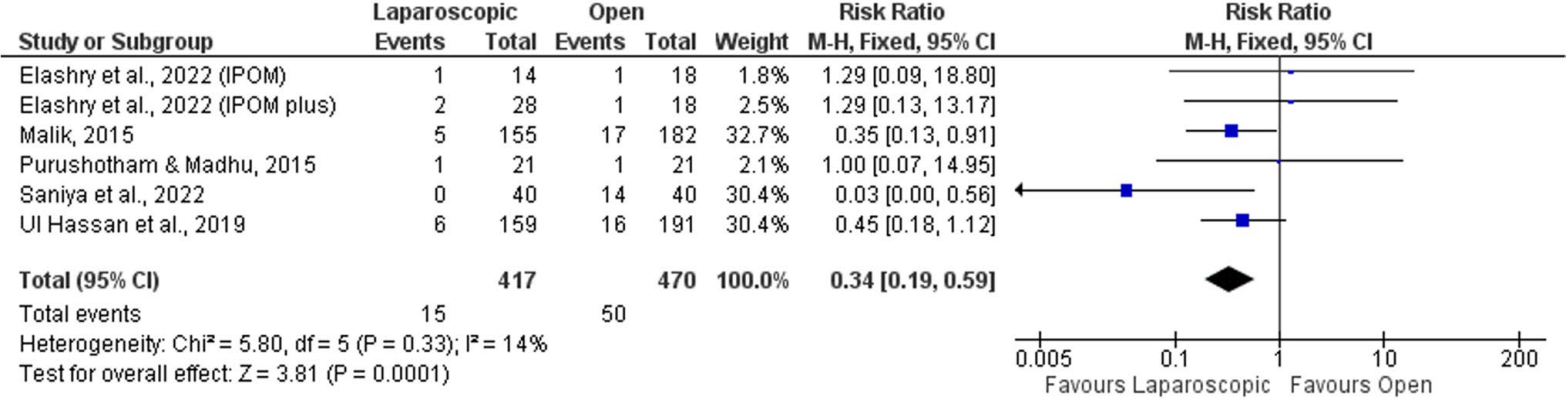
Forest plot of local seroma

There were five studies [51, 52, 56, 57, 59] measuring the occurrence of local seroma, with meta-analytical results indicating that local seroma was 66% less probable to develop in the laparoscopic approach in comparison with the open approach (RR = 0.34; 95%CI = 0.19–0.59; *p* < 0.001; Fig. 7), with low heterogeneity detected (*I*^2^ = 14%).

#### Postoperative pain

**Fig. 8:**
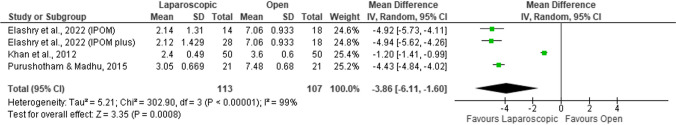
Forest plot of postoperative pain

Six studies assessed postoperative pain [51, 52, 54, 56, 57, 59]. We were able to meta-analytically assess three studies [51, 52, 54]. Regarding this outcome, the laparoscopic approach showed significant benefits when compared with the open approach (MD =  − 3.86, 95%CI =  − 6.11; − 1.60; *p* < 0.001; Fig. 8). However, the results were substantially heterogeneous (*I*^2^ = 99%; *p* < 0.001).

Nevertheless, all the included studies showed that the pain was significantly lower in the laparoscopic repair group when compared to the open repair group. In two of these studies [51, 54], postoperative pain was evaluated more than once, and only the 24-h measure was considered in the analysis. In one of the studies, Khan et al*.* [54], in addition to the 24-h measure, a 2-h measurement was performed and revealed that the postoperative pain was significantly less in the laparoscopic repair group. In the other study, Purushotham and Madhu [51], in addition to the 24-h measure, a 6-h measurement was performed, and there were no significant differences between the laparoscopic repair group and the open repair group.

Regarding the studies we were not able to include in the meta-analysis, Saniya et al*.* [57] was excluded from the analysis because they assessed the number of patients with postoperative pain and not the score of pain (0–10). In this study, the number of patients with postoperative pain was significantly higher in the open repair group.

The studies of Malik [56] and Ul Hassan et al*.* [59] only evaluated prolonged pain (> 4 months) and in consequence were excluded from the analysis. In both studies, the laparoscopic repair group had fewer cases of prolonged pain when compared with the open repair group.

#### Duration of the surgery (minutes)

The duration of the surgery was described in all the studies. Nonetheless, two of the studies [56, 59] were excluded from the analysis because they assessed the number of patients in three subgroups of time (40–60 min; 61–90 min; > 90 min) and did not provide information on the mean duration of the surgery. The majority of the open surgeries were included in the 40–60 min subgroup, and the majority of laparoscopic surgeries were included in the 61–90 min subgroup. Two further studies [55, 58] were excluded from the analysis due to the absence of reported spread measures. In these studies, the mean surgery duration was significantly less in the laparoscopic repair group when compared with the open repair group.

**Fig. 9:**
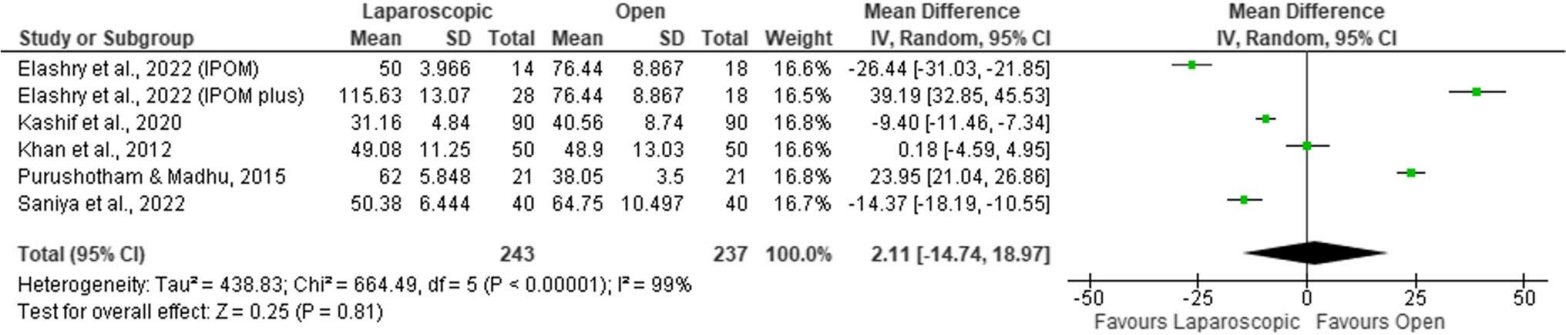
Forest plot of duration of the surgery

The remaining four studies [51–54, 57] were included in the meta-analysis; however, the results were not statistically significant (MD = 2.11; 95%CI =  − 14.74, 18.97; *p* = 0.810; Fig. 9) and were substantially heterogeneous (*I*^2^ = 99%; *p* < 0.001). Khan et al*.* [54] found no significant differences between the groups. Elashry et al*.* [52] found significant differences in both IPOM (intraperitoneal onlay mesh) and IPOM plus group when compared with the open repair group: the duration of surgery was significantly lower in the IPOM group versus the open repair group and significantly higher in the IPOM plus group when compared with the open repair group. Kashif et al*.* [53] and Saniya et al*.*[57] concluded that the duration of surgery was significantly lower in the laparoscopic group. And, finally, Purushotham and Madhu [51] established that the duration of surgery was significantly higher in the laparoscopic group.

#### Length of hospital stay (days)

The length of hospital stay was described in all the studies. Nonetheless, two of the studies [56, 59] were excluded from the analysis because they subdivided the patients into two groups (patients with and without complications); in both subgroups, the laparoscopic repair was associated with less time in the hospital post-surgery and the patients with complications had, in general, more time of hospital stay.

**Fig. 10:**
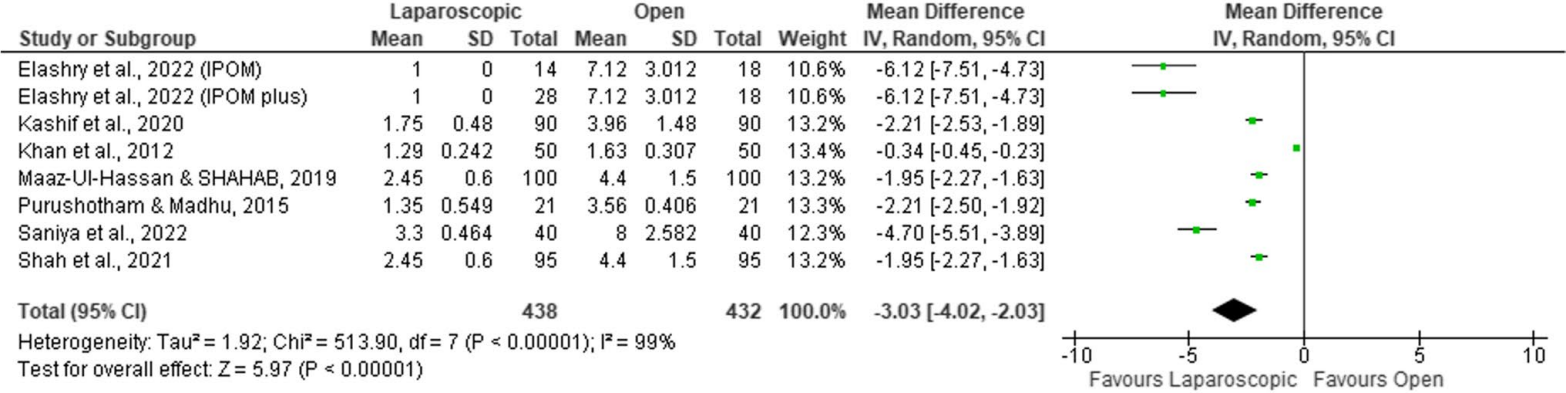
Forest plot of length of hospital stay

A total of seven studies were included in the analysis [51–55, 57, 58] with meta-analytical results indicating that the laparoscopic approach was associated with an average decrease of 3 days in the length of hospital stay (MD =  − 3.03; 95%CI =  − 4.02, − 2.03; *p* < 0.001; Fig. 10); nonetheless, the results were substantially heterogeneous (*I*^2^ = 99%; *p* < 0.001). Furthermore, all the studies included in the analysis showed that the length of hospital stay was significantly lower in the laparoscopic repair group versus the open repair group.

#### Time until return to work (days)

**Fig. 11:**
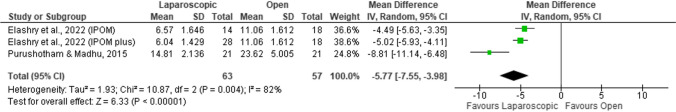
Forest plot of time until return to work

Two studies [51, 52] evaluated the time needed until a patient could return to work. Regarding this outcome, the laparoscopic approach showed significant benefits and was associated with an average decrease of 6 days in the time until return to work (MD =  − 5.77; 95%CI =  − 7.55, − 3.98; *p* < 0.001; Fig. 11). However, high heterogeneity was found (*I*^2^ = 82%; *p* = 0.004). Nonetheless, all the included studies indicated that the time needed until a patient could return to work was significantly lower in the laparoscopic repair group when compared to the open repair group.

#### Leave-one-out sensitivity analysis

A sensitivity analysis was performed for postoperative pain, duration of surgery, length of hospital stay, and time until return to work. Most of the sensitivity analyses failed to identify differences between studies.

Regarding postoperative pain, the exclusion of Khan et al*.* [54] was associated with a reduction of heterogeneity from 99 to 9%, and the laparoscopic approach showed significant benefits when compared with the open approach (MD =  − 4.64; 95%CI =  − 4.98, − 4.29; *p* < 0.001; *I*^2^ = 9%; *p* = 0.330).

Additionally, the exclusion of Purushotham and Madhu [51] was related to a complete reduction of the heterogeneity in time until the return to work. The laparoscopic repair showed an average reduction of 5 days in the time until return to work in comparison to open repair (MD =  − 4.81; 95%CI =  − 5.53, − 4.10; *p* < 0.001; *I*^2^ = 0%; *p* = 0.480).

## Discussion

Recent studies concluded that primary and incisional ventral hernias were statistically significantly different for almost all patients regarding hernias, surgical, and postoperative characteristics. Furthermore, they say that given these differences, data on primary hernias, and incisional hernias should not be pooled in studies reporting on hernia repair [5, 60].

Regarding these new findings, we conducted this systemic review and meta-analysis that, unlike similar previous systematic reviews [4, 9, 34, 61], to the best of our knowledge, was the first one to solely evaluate patients with the diagnosis of primary ventral hernia.

A total of nine studies were included in the meta-analysis. The meta-analysis results revealed that the overall hernia recurrence, local infection, wound dehiscence, and local seroma were significantly less likely to be present in a patient who underwent the laparoscopic approach. These findings go alongside the expected based on previous studies. Although the heterogeneity between studies was non-existent or mild, the results should be analyzed carefully as all the included studies had some or a high risk of bias and did not specify how the outcomes were evaluated.

The data on postoperative pain, length of hospital stay, and time until return to work were substantially heterogeneous. However, the laparoscopic approach seemed beneficial in all the included studies.

Regarding the duration of surgery, there was a lot of controversy across the studies. This heterogeneity could be justified by the presence of a learning curve in the laparoscopic repair, by the time required for handling the mesh, or by adversities that could be found during the surgery and could influence the duration of the surgery. The authors of Elashry et al*.* [52] concluded that the significant difference in prolongation of the time in IPOM plus was due to handling the mesh intra-peritoneally, but with experience, this difficulty could be overcome. Al-Mulhim et al*.* [62] and Nijas et al*.* [63] also concluded that the time for laparoscopic repair decreased with the progress in the learning curve.

Assessing the limitations of this systematic review and meta-analysis, firstly, it should be noted that the included studies, which were all randomized controlled trials, had some concerns regarding the allocation of the patients, and the methods used were not well described which could lead to serious bias.

Secondly, this systematic review and meta-analysis included a small number of studies in which the type of hernia and surgical approach varied between studies. In these cases, a subgroup analysis could be beneficial, due to the small number of included primary studies that was not feasible.

Also, the included studies had, in general, small sample sizes, and two studies [56, 59] had missing data and a combined total of 23 laparoscopies that were converted to open surgeries and analyzed as such (intention-to-treat analysis). These characteristics of primary included studies could have some implications on the interpretation of the results because they could lead to heterogeneity and bias.

Furthermore, the included studies had a follow-up period of 2 years or less, and, in the majority of studies, the hernia size was 4 cm or less. These could lead to a smaller number of reported hernia recurrences and other complications. Additionally, hernia size could be a confounder in some of the outcomes, such as seroma and hernia recurrence.

In order to identify if any individual study was associated with higher heterogeneity, a leave-on-out sensitivity analysis was made. Regarding postoperative pain, the exclusion of Khan et al*.* [54] was associated with a reduction of heterogeneity from 99 to 9%. These could be related to the fact that Khan et al*.* [54] included epigastric, umbilical, and paraumbilical hernias, while Elashry et al*.* [52] only included paraumbilical hernias and Purushotham and Madhu [51] umbilical and paraumbilical hernias.

Moreover, the exclusion of Purushotham and Madhu [51] was related to a complete reduction of the heterogeneity in time until the return to work, which could be justified by the different hernia types included in the individual studies and the small number of primary studies included in the meta-analysis.

Regarding the potential influence of publication bias on the results of this systematic review and meta-analysis, although it was difficult to evaluate its specific impact due to the small number of included primary studies in each outcome, it can be considered small. An extensive literature search was executed; therefore, it is unlikely that important randomized controlled trials were not identified by the initial search.

The overall treatment of primary ventral hernias appeared to be more beneficial in terms of clinical outcomes in the laparoscopic approach. Nonetheless, the published guidelines only recommend the laparoscopic approach in specific patients [7, 8].

In this regard, it is worth mentioning that, although our meta-analysis suggests that laparoscopic surgery in primary ventral hernia repair is beneficial and advantageous, this study is not free of limitations and some aspects (duration of surgery, hernia recurrence, and post-operative pain) need to be studied in more detail to help direct future research and development of specific guidelines for primary ventral hernia repair as an independent entity.

## Conclusion

Currently, the results of available studies for the treatment of patients with primary ventral hernia remain somewhat controversial and with low-quality evidence. Even though all the studies are randomized controlled trials, the majority have a high risk of bias, few results, scarce samples, and few outcomes assessed and don’t have well-defined protocols (no sample size calculation, no primary outcome defined, and do not specify the methods used to assess the outcomes).

Though the available evidence is weak and the existing studies have low quality, we assume that the laparoscopic approach of the primary ventral hernia repair seems beneficial concerning hernia recurrence, local infection, wound dehiscence, and local seroma. Additionally, it seemed to improve the postoperative pain, length of hospital stay, and time until return to work. However, this is yet to be proven.

Further and larger studies are needed, namely randomized controlled trials, methodologically well executed, with an adequate number of participants, and a sufficient follow-up period before definitive conclusions on the true value of this procedure can be derived in order to allow confirmation of these results.

## Data Availability

All the data supporting the findings of this study are available from the authors upon reasonable request.
